# Orthodox and Unorthodox Medicine

**Published:** 1902-10-11

**Authors:** 


### Orthodox and Unorthodox Medicine.

In his Presidential Address to the American Medical Association, Dr. Wyeth, of New York, propounded the view that the time has come for some modifications of the rules of conduct laid down in the Code of Ethics which was adopted by the respected founders of that Association and still remains in force among its members. He referred more particularly to a question which in America intrudes much more into professional life than it does with us, namely, as to the relations to be maintained between orthodox medicine and the various dissenting creeds of medical practice or belief. He pointed out that one section of the rules of conduct in the Code of Ethics to which they all subscribe "forbids a member of the regular profession to act upon a board of examiners which has to pass upon the legal qualifications of persons not graduates of regular medical colleges." Yet the civil statutes of 38 States require these boards, which are composed in great part of members of the Association, to examine, pass, certify, and license to practise "homœopaths, eclectics, and other subdivisions of medical practice." Moreover, as he says, in certain States the law expressly states "Distinctions shall not be made between applicants because of the different systems or schools of practice that may be chosen," and he points out, as a condition to be regretted, and if possible to be altered, that "in almost all the States and territories regular physicians are compelled by the laws of the State in which they reside to disobey the injunctions of this section of the Code of Ethics." Undoubtedly this is a position which brings upon the carpet many greatly cherished articles, if not of faith at least of conduct; and Dr. Wyeth seems to hint that as the mountain will not come to Mahomet —as the State law will not conform with the Code —Mahomet must fain go to the mountain, and the Code must be altered. With us, fortunately, these are not such burning questions as they are elsewhere. Whether this is due to the fact that the "opathies" are voiceless, not having medical schools of their own, or whether, as is more probable, the extraordinary readiness shown by English practitioners to adopt any form of treatment which is found useful sucks the life blood out of medical dissent, leaving its professors mere sticklers for dry doctrine, the fact remains that unorthodox medicine in England is unable to present a solid front as it does in America, or to become a real problem apart from the general tendency of modern fashion to run to fads.

One must not forget, however, that if the professors of any kind of medical tomfoolery found it worth their while to set up a properly equipped medical school, and, while teaching the ancillary sciences correctly, were to confine "treatment" to some such hocus pocus, for example as the laying on of hands or the administration of infinitesimal doses, there would be but little to prevent the pupils of such a sect becoming fully qualified practitioners, for it is distinctly stated in the Medical Act that "In case it shall appear to the General Council that an attempt has been made by any body, entitled under this Act to grant qualifications, to impose upon any candidate offering himself for examination an obligation to adopt or refrain from adopting the practice of any particular theory of Medicine or Surgery, as a Test or Condition of admitting him to examination or of granting a certificate, it shall be lawful for the said Council to represent the same to Her Majesty's Most Honourable Privy Council, and the said Privy Council may thereupon issue an injunction to such Body so acting, directing them to desist from such practice; and in the event of their not complying therewith, then to order that such Body shall cease to have the power of conferring any right to be registered under this Act so long as they shall continue such practice."

Whether the General Council would be easily persuaded to draw the attention of the Privy Council to such a matter we cannot tell, but we can hardly believe that a clause so obviously intended to defeat the tyranny of effete ideas would be allowed to stand idle if there were occasion for its use; and we can but believe that the reason why medical dissent remains, if one may so describe it, "disestablished" in England, is that orthodox medicine has been always ready (outrageously and foolishly ready according to some) to take to itself and to use for all that it is worth any method of treatment which can show good results. The non-existence of unorthodox medical schools in England is then the highest possible compliment to the liberality of orthodoxy.

